# Optimization of Fermentation Process for New Anti-Inflammatory Glycosylceramide Metabolite from *Aspergillus* sp.

**DOI:** 10.3390/metabo14020099

**Published:** 2024-01-31

**Authors:** Yung-Husan Chen, Qiaoqiao Zhu, Jingyi Li, Rong Yang, Jingwen Zhang, Minxin You, Lianzhong Luo, Bingye Yang

**Affiliations:** 1 Xiamen Key Laboratory of Natural Products Resources of Marine Medicine, Xiamen Medical College, Xiamen 361023, China; zhuqq@stu.njmu.edu.cn (Q.Z.); 202005440053@xmmc.edu.cn (J.L.); 201400080002@xmmc.edu.cn (R.Y.); 202102460027@xmmc.edu.cn (J.Z.); 202205480025@xmmc.edu.cn (M.Y.); llz@xmmc.edu.cn (L.L.); 2 Fujian Provincial University Marine Biomedical Resources Engineering Research Center, Xiamen Medical College, Xiamen 361023, China

**Keywords:** *Aspergillus* sp., glycosylceramid, orthogonal experiments, fermentation optimization, response surface optimization

## Abstract

A novel ceramide compound, named Aspercerebroside A (AcA), was successfully isolated from the ethyl acetate layer of the marine symbiotic fungus *Aspergillus* sp. AcA exhibited notable anti-inflammatory activity by effectively inhibiting the production of nitric oxide (NO) in RAW 264.7 cells at concentrations of 30 μg/mL and 40 μg/mL, offering a promising avenue for the treatment of inflammatory diseases. To optimize the yield of glycosylceramide (AcA), a series of techniques, including single-factor experiments, orthogonal experiments, and response surface optimization, were systematically employed to fine-tune the composition of the fermentation medium. Initially, the optimal carbon source (sucrose), nitrogen source (yeast extract powder), and the most suitable medium salinity (14 ppt) were identified through single-factor experiments. Subsequently, orthogonal experiments, employing an orthogonal table for planning and analyzing multifactor experiments, were conducted. Finally, a mathematical model, established using a Box–Behnken design, comprehensively analyzed the interactions between the various factors to determine the optimal composition of the fermentation medium. According to the model’s prediction, when the sucrose concentration was set at 37.47 g/L, yeast extract powder concentration at 19.66 g/L, and medium salinity at 13.31 ppt, the predicted concentration of glycosylceramide was 171.084 μg/mL. The experimental results confirmed the model’s accuracy, with the actual average concentration of glycosylceramide under these conditions measured at 171.670 μg/mL, aligning closely with the predicted value.

## 1. Introduction

As valuable sources of novel pharmaceuticals and bioactive compounds, secondary metabolites have been generated by various species within the *Aspergillus* genus, and their prominence has been steadily increasing. Among the common marine fungi belonging to the *Aspergillus* genus are *Aspergillus* nidulans, *Aspergillus* oryzae, *Aspergillus* versicolor, *Aspergillus* nidurans, *Aspergillus* carneus, *Aspergillus* wentii, and others [1,2]. These fungal cells have the capacity to produce a diverse array of secondary metabolites, including alkaloids, polyketides, terpenes, peptides, and a variety of other categories [3].

Chemical analysis has revealed that ceramides consist of long-chain bases (LCBs) and long-chain fatty acid amides (FABs), linked together by an amide bond [4]. Biological analysis has shown that ceramides and their glycosylated counterparts, cerebrosides, can exhibit a wide range of biological activities, including cytotoxicity, antifungal properties, immunostimulation, and immunosuppressive effects [5]. These ceramides and cerebrosides have been extracted from various marine invertebrates, including starfish, sea anemones, sponges, corals, ascidians, and bryozoans, and from some terrestrial plants as well.

Glycosylceramides have progressively found applications in various aspects of daily life. Their potential properties for cancer prevention, immunostimulation, and anticancer effects have been demonstrated. Consequently, they have been employed as functional foods or pharmaceuticals to enhance human physiological well-being. Clinical research has highlighted age-related declines in glycosylceramide levels. In particular, glycosylceramide supplementation has shown the potential to effectively slow down the aging process of facial skin and hand skin [6,7].

Glycosylceramides were discovered to play a pivotal role as structural precursors for various sphingolipids in previous studies. They could be modified to produce hydrophilic head groups, resulting in a range of compounds, including glycosphingolipids, sphingomyelins, and glycosylceramides. These substances, identified as critical constituents of biological membranes, contributed to the formation of diverse complex sphingolipids, gangliosides, and cerebrosides within the hydrophobic backbone of the body [8]. Glycosylceramides are typically localized in specific regions of cell membranes, where membrane integrity was preserved, and they become involved in cell signaling through influencing receptor oligomerization, signal amplification, or modification of cell signals. Moreover, owing to their hydrophobic nature, glycosylceramides have been identified as essential components of the skin’s stratum corneum membrane, where they aid in the establishment of the skin’s permeability barrier and play a pivotal role in skin hydration [9].

The research highlights the significant role played by long-chain fatty acid amides (FABs) in skin barrier function, particularly in the formation of lipid envelopes within keratinocytes [10]. Reduced sweating ability is often experienced by individuals with atopic dermatitis (AD) due to epidermal barrier dysfunction, resulting in a marked reduction in glycosylceramides esterified with linoleic acid (LA) within the stratum corneum. This leads to sweat duct blockage and prolonged sweating latency. The elevation of glycosylceramide levels in the stratum corneum has been shown to ameliorate epidermal barrier dysfunction, reduce sweating latency, and enhance stratum corneum function [11,12].

Glycosylceramides, serving as novel and highly effective moisturizers, have found extensive application in skincare products. They effectively mitigate moisture and lipid loss in the epidermis and dermis by stabilizing glycosylceramides on cell membranes, thereby delivering exceptional moisturization [13]. Additionally, they enhance skin elasticity, resistance, and slow the aging process of the skin [14]. However, due to their unique molecular structure, glycosylceramides possess a high melting point and low solubility, making them unsuitable for direct use as components in skincare products [15]. To address this challenge, numerous cosmetics companies are actively researching and developing innovative raw materials such as glycosylceramide liposomes and glycosylceramide encapsulates. These glycosylceramide-based skincare products have gained popularity among consumers.

In this study, a fungus was isolated from *Dendronephthya* sp. on Xiongdiyu Island, Dongshan Island, Zhangzhou City, Fujian Province, and was preliminarily identified as *Aspergillus* sp. From the ethyl acetate extract of this fungus, a glycosylceramide compound named AcA was isolated. Through spectroscopy and chemical analysis, it was discovered that AcA inhibited the production of nitric oxide (NO) in RAW 264.7 macrophage cells stimulated by lipopolysaccharide (LPS) at concentrations of 30 μg/mL and 40 μg/mL, demonstrating its anti-inflammatory properties. Building upon single-factor experiments, the Design-expert 8.0.6.1 software was utilized to establish a mathematical model based on the Box–Behnken design to optimize the fermentation medium composition of this fungus. On the basis of the results, response surface methodology (RSM) was applied to optimize the optimized glycosylceramide condition. The objective was to enhance the content of glycosylceramide, particularly AcA, in the secondary metabolites of this fungal strain, thus laying the experimental groundwork for large-scale industrial fermentation production of glycosylceramides. Through the successful optimization of the fermentation medium for AcA production by Aspergillus sp., an extract rich in glycosylceramides from marine filamentous fungi was obtained.

## 2. Materials and Methods

### 2.1. Chemicals and Equipment

The chemicals and reagents used in this study included methanol, acetic acid, ethyl acetate, glucose, sucrose, glycerol, and soluble starch (purchased from Xilong Scientific Co., Ltd., Guangzhou, China); chromatographic methanol (purchased from Guangzhou Tongyuan Chemical Technology Co., Ltd., Guangzhou, China); marine saltwater (provided by Jiangxi Yantong Technology Co., Ltd., Ningdu, China); N-acetylglucosamine (purchased from Shanghai Maclin Biochemical Technology Co., Ltd., Shanghai, China); peptone, yeast extract powder, potato glucose broth dry medium, and potato glucose agar dry medium (purchased from Guangdong Huanke Microbial Science and Technology Co., Ltd., Guangzhou, China); silica gel (200–300 mesh, Qingdao Marine Chemical Co., Ltd., Qingdao, China); Sephadex LH-20 gel (purchased from Nanjing Yuanbaofeng Medical Technology Co., Ltd., Nanjing, China); a laminar flow cabinet (model SW-CJ-2FD, Suzhou Antai Air Technology Co., Ltd., Suzhou, China); an autoclave sterilizer (model GR85DR, Zhengzhou Instrument Co., Ltd., Zhengzhou, China); a biochemical incubator (model SPX-250B-Z, Shanghai Boyuan Industrial Co., Ltd. Medical Equipment Factory, Shanghai, China); an orbital shaker (model ZQZY-CGF8, Shanghai Zhichu Instrument Co., Ltd., Shanghai, China); an ultrasonic cleaning machine (model DS-080S, Dongsen Washing Technology Co., Ltd., Zhengzhou, China); an Abbott salinometer (Wuhan Zhongxia Medical College undergraduate thesis design Hongtu measurement instrument Co., Ltd., Wuhan, China); a rotary evaporator (model RE212-B, Shanghai Guigu Technology Development Co., Ltd.); and an Agilent 1260 high-performance liquid chromatography system (Agilent Technologies, Inc., Santa Clara, CA, USA). Ultraviolet (UV) spectra were obtained using a Shimadzu UV-1800 UV/vis spectrophotometer. IR data were acquired by a Bruker TENSOR27 spectrophotometer. High resolution ESIMS and MS/MS experiments were carried out on a Q-Exactive (Thermo Scientific, Waltham, MA, USA) mass spectrometer. NMR spectra were determined on a BRUKER AVANCE III 500 MHz NMR spectrometer at 298.0 K equipped with 5 mm probes (500 MHz for ^1^H and 125 MHz for ^13^C NMR, Bruker BioSpin, Billerica, MA, USA). The ^1^H and ^13^C NMR spectrum were referenced to the solvent peaks for DMSO-d6. HPLC purification was performed on a PURIFLASH^®^ 4250 equipped with QuikSep-DAC50 series DAC-50 dynamic axial compression column system (YMC-Triart C18 S-10 μm particles, H&E Co., Ltd., Beijing, China). All solvents were purchased from Merck KGaA (Darmstadt, Germany), if not mentioned.

### 2.2. LC-ESI-MS/MS Analysis

One μL of AcA content was injected into the LCESI-MS/MS system (LC system: Waters CapLC; MS system: nanoACQUITY/QExactive). The flow rate of the LC system was 6 μL/min through the column system (Waters Atlantis dC18″, 3 μm, 75 μm × 50 μm column) and the flow rate eventually was reduced to 0.3 μL/min by the splitter before entering into chromatographic column system. The solvent gradient system consisted of 5–100% methanol. The solvent increased from 5% to 100% in 4 min and then equilibrated for 1 min.

Mass spectrometry was performed in +ESI ionization mode with voltage of 4.50 kV and desolvation temperature of 290 °C. Triple quadrupole detection was controlled at an accelerating voltage of 9.1 kV, cone voltage of 100 V, and impact energy of 10 eV. The scanning range of mass spectrometry was monitored between *m/z* 600 and 800. The selected reaction model (SRM) mode was used to quantify the AcA contents. The ion chromatography peak area of the product ion was compared with the standard, and then the contents of AcA in the *Aspergillus* sp. extracts were calculated. The mass spectrometer tuning and calibration standard was [Glu]-Fibrinopeptide B (Sigma Chemicals, Kewdale, WA, USA).

### 2.3. Strain and Fermentation Conditions

The marine fungus *Aspergillus* sp., isolated from the waters of Dongshan Island, Zhangzhou City, Fujian Province, is currently preserved in the Guangdong Microbial Culture Collection Center (registration number GDMCC61933). Seed culture media included potato dextrose agar dry medium (PDA) and potato dextrose broth dry medium (PDB). Fermentation media comprised carbon source single-factor fermentation medium, nitrogen source single-factor fermentation medium, and response surface-optimized fermentation medium.

### 2.4. Strain Propagation

Propagation of the strain involved transferring a 1 cm^2^ single colony from a successfully passaged culture onto potato dextrose agar dry medium (PDA) and incubating at room temperature for 1–2 months under sealed conditions.

### 2.5. Organic Solvent Extraction

Sterile extraction was carried out using a mixed solvent A (ethyl acetate: methanol: acetic acid = 80:15:5). Repeated extraction was performed to obtain secondary metabolites, and rotary evaporation was used to concentrate the extract, resulting in a weight of M0 (134.59 g).

A mixed solvent B (ethyl acetate: water = 1:1) was used for liquid–liquid extraction of the concentrated extract, retaining the ethyl acetate layer. Rotary evaporation was then employed to obtain a weight of M (52.11 g).

### 2.6. Silica Gel Column Chromatography

The ethyl acetate layer concentrate (DS142-1) was passed through a normal-phase silica gel column (200–300 mesh) using the following elution conditions: 100% petroleum ether → petroleum ether: ethyl acetate = 90:1 → petroleum ether: ethyl acetate = 50:1 → petroleum ether: ethyl acetate = 30:1 → petroleum ether: ethyl acetate = 10:1 → petroleum ether: ethyl acetate = 5:1 → petroleum ether: ethyl acetate = 3:1 → petroleum ether: ethyl acetate = 1:1 → 100% petroleum ether → 100% methanol. This yielded 18 components.

A portion of DS142-1-18-2 (2.1719 g) was passed through a normal-phase flash column (300–400 mesh silica gel) using the elution conditions: dichloromethane: methanol = 36:1 → dichloromethane: methanol = 32:1 → dichloromethane: methanol = 30:1 → dichloromethane: methanol = 20:1 → 100% methanol. This resulted in five components: DS142-1-18-1 (20.1 mg), DS142-1-18-2 (17.5 mg), DS142-1-18-3 (1.0511 g), DS142-1-18-4 (200 mg), DS142-1-18-5 (880 mg).

DS142-1-18-2-3 was subjected to liquid-phase analysis and purification using a Welch Ultimate^®^ XB-C18 column (5 μm, 10 × 250 mm) with 100% methanol as the eluent, monitored at 210 nm and 254 nm, and a flow rate of 2 mL/min. This process yielded compound 1 (2.3 mg).

#### 2.6.1. Optimization of Culture and Fermentation Conditions

Seed Cultivation: The frozen strain suspension stored at −80 °C was gradually thawed, and a small amount of the thawed culture was spread and activated on solid PDA medium within a laminar flow hood. After incubating at 25 °C for 72 h, spores from the activated strain were collected. These spores were then inoculated into 250 mL triangular flasks containing 100 mL of PDB liquid culture medium and incubated on a light-shaking incubator at 180 rpm for 24 h to obtain the seed culture.

Seed Culture Medium Fermentation Conditions: Salt concentration in the culture medium: 15 ppt; pH: 7.2–7.4; temperature: 25 °C; shaking speed: 180 rpm; fermentation time: 24 h.

Optimization of Culture Medium Fermentation Conditions: pH: 7.2–7.4; temperature: 25 °C; shaking speed: 150 rpm; fermentation time: 72 h.

#### 2.6.2. Sample Extraction

The fermentation broth was filtered and separated using four layers of gauze to obtain mycelial balls and clear liquid. The clear liquid was subjected to extraction with an equal volume of ethyl acetate, while the mycelial balls were subjected to ultrasound-assisted extraction 2–3 times using a mixed solution system of ethyl acetate: methanol: acetic acid = 80:15:5. After filtering and separating the mycelial balls and the extract, the extract was further extracted with ethyl acetate twice, and the combined ethyl acetate extracts were concentrated using rotary evaporation to obtain the crude extract. The weight of the crude extract was accurately measured and recorded.

Dry loading was performed using 1–1.5 times the mass of diatomaceous earth as an adsorbent, and 1–2 mL of methanol was used to dissolve the sample. The two components were mixed thoroughly to ensure that diatomaceous earth fully adsorbed the sample. After the organic reagents evaporated, the sample was dried at 45 °C for 3 h in an oven. Subsequently, a C18 SPE reverse-phase solid-phase extraction column was used for initial separation, retaining the extract under 100% methanol elution conditions. After rotary evaporation, 1 mL of methanol was used for ultrasonic dissolution, and the sample was filtered through a 0.22 μm filter membrane to obtain the final sample for HPLC analysis.

#### 2.6.3. Determination of Glycosylceramides Content

Chromatographic Detection Conditions: Ultimate XB-C18 column: 4.6 × 250 mm, 5 µm; mobile phase: ultrapure water–methanol, flow rate: 0.8 mL/min; detection wavelength: 210 nm; injection volume: 8 μL; temperature: 26 °C; methanol elution for 20 min.

Construction of Standard Curve: Precisely weigh 10 mg of glycosylceramide standard into a 10 mL volumetric flask, dissolve it in methanol, shake it well, and bring it to a standard solution of 1 mg/mL. Subsequently, prepare a series of glycosylceramide standard solutions with concentrations of 200 μg/mL, 100 μg/mL, 75 μg/mL, 50 μg/mL, 25 μg/mL, and 10 μg/mL from the stock solution, and analyze them in triplicate using HPLC. Finally, construct a linear standard curve with peak area as the independent variable and glycosylceramides concentration as the dependent variable. The equation for the standard curve is y = 3.5932x − 3.6172, R^2^ = 0.9996, where x represents peak area and y represents glycosylceramides concentration.

#### 2.6.4. Determination of NO Content

An in vitro NO production inhibition experiment was conducted following a method reported in the literature [16]. The experiment was divided into four groups: normal group, LPS group, Ac A group (30 ug/mL, 40 ug/mL. RAW 264.7 cells (5 × 10^6^ cells in a 96-well plate) were incubated for 6 h in a humidified incubator (37 °C, 5% CO_2_). After incubation, the culture medium for the normal group was replaced with serum-containing medium. The LPS group was treated with LPS (0.2 ug/mL), and the two Ac A groups were treated with Ac A (30 ug/mL and 40 ug/mL) and LPS (0.2 ug/mL), respectively, followed by a 24 h incubation. Equivalent supernatants from RAW 264.7 cell cultures were collected and subjected to NO activity assays using a NO production inhibition assay kit (Cat NO S00215, Beyotime Biotechnology, Nantong, China).

#### 2.6.5. Single-Factor Impact Experiments

Effect of Different Carbon Sources on glycosylceramides Production: Different carbon sources (glucose/sucrose/soluble starch/glycerol) at a concentration of 40 g/L were used as carbon sources in the fermentation medium, with 10 g/L of peptone as the nitrogen source for single-factor experiments. Each experimental group was repeated in triplicate. After fermentation, glycosylceramides were extracted from the metabolic products and analyzed by HPLC. The concentration of glycosylceramides under different carbon source conditions was determined using the standard curve. Significant differences in glycosylceramides production under different carbon source conditions were analyzed using SPSS software (analysis method: LSD, *p* < 0.01).

Effect of Different Nitrogen Sources on glycosylceramides Production: Different nitrogen sources (peptone/yeast extract/N-acetylglucosamine) at a concentration of 10 g/L were used as nitrogen sources in the fermentation medium, with 40 g/L of sucrose as the carbon source for single-factor impact experiments. Each experimental group was repeated in triplicate. After fermentation, glycosylceramides in the metabolic products were qualitatively and quantitatively determined by HPLC. The concentration of glycosylceramides under different nitrogen source conditions was determined using the standard curve. SPSS software was used to analyze the results (analysis method: LSD, as mentioned earlier), and the nitrogen source that resulted in the highest glycosylceramides content was identified.

Effect of Different Medium Salinities on glycosylceramides Production: Using 40 g/L sucrose as the carbon source and 10 g/L yeast extract as the nitrogen source, the salinity of the fermentation medium was varied to 6 ppt, 14 ppt, 22 ppt, and 30 ppt to investigate the effect of different medium salinities on glycosylceramide production by the strain. Each salinity condition was repeated in triplicate. After fermentation, combining the qualitative and quantitative results obtained by HPLC with the standard curve, the optimal medium salinity for glycosylceramide production by the strain was determined using SPSS-22.0 software.

#### 2.6.6. Orthogonal Experimental Design

Based on the results of the single-factor experiments for various influencing factors and their levels, *Aspergillus* sp. was cultured in a fermentation medium containing 45 g/L sucrose, 8 g/L yeast extract, and an appropriate amount of marine salt. An orthogonal table L9 (34) was used according to the selected influencing factors and the number of levels.

#### 2.6.7. Response Surface Analysis for Optimization of Fermentation Medium

Using the selected factors of sucrose, yeast extract, and medium salinity, with glycosylceramides production as the response value, a three-factor, three-level experiment was established using the Box–Behnken design in the response surface design software Design-expert 8.0.6.1. This was performed to optimize the fermentation medium formula for *Aspergillus* sp. The factors and levels for the response surface experiments are listed in Table 1.

#### 2.6.8. Model Validation

The optimal culture medium formula for *Aspergillus* sp. from marine filamentous fungi, as determined by the Box–Behnken design method, was experimentally validated to assess the reliability of the model and analyze the final optimization results.

## 3. Results

### 3.1. Structural Analysis

The crude extracts from *Aspergillus* sp. were separated by a silica gel column and purified with Sephadex LH-20 and high-performance liquid chromatography to obtain AcA. The structure of the isolated compound was established by analyzing its spectroscopic data including UV, IR, 1D (^1^H and ^13^C), and 2D NMR (^1^H-^1^ H COSY, HMQC, HMBC, NOESY) and HR-ESI-MS] and comparing the obtained data with what was reported in refs [17,18,19,20].

AcA was isolated as a colorless amorphous powder. The HR-ESI-MS (positive ion mode) *m*/*z*: 768.56 [M+H]^+^ and the ^13^C-NMR data suggested a molecular formula of C_44_H_81_NO_9_. The infrared spectrum of AcA showed absorption bands of hydroxy (3310 cm^−1^) and carbonyl amide (1626 cm^−1^ and 1532 cm^−1^) functional groups. The ^1^H and ^13^C-NMR spectra of AcA (Table 2) showed characteristic signals of glycoside derivatives (also known as cerebroside). These signals included anomeric protons at ***δ*_H_** 4.11 (d; *J* = 7.75), carbon oxides (sugar portion) of H-1″ and ***δ*_C_** 61.06–76.88, carbonyl carbons of H-1′ and ***δ*_C_** 172.01, C-1′ (amide bond) and terminal methyl protons of ***δ*_H_** 0.85 (t; *J* = 6.875), and methylene protons of ***δ*_H_** 1.23–3.97 (m) (long-chain hydrocarbons). The glucose ^13^C-NMR signals were ***δ*_C_** 61.06 (C-6″), 70.01 (C-4″), 73.39 (C-2″), 76.54 (C-3″), 76.88 (C-5″), 103.50 (C-1″). The trans geometry of the C-8 double bond was established by the chemical shifts of the methyl groups attached to the C-9, which was ***δ*_C_** 15.72 (C-18).

**Figure 1:**
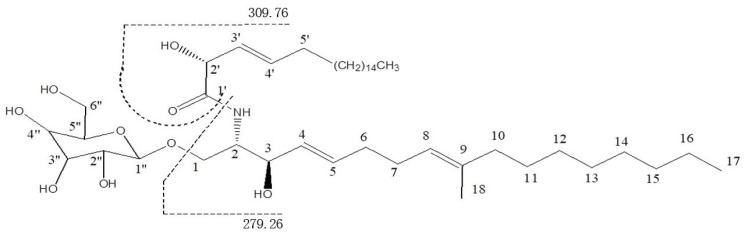
The chemical structural formula of Aspercerebroside A.

The length of the sphingoid long-chain base (LCB) and the amide-linked long-chain fatty acid base (FAB) were suggested as 17 and 20 carbons, respectively. The fragment ions at *m*/*z* 310.76 [FAB+H]^+^ confirmed the length of the amide-linked long-chain fatty acid (FAB). Moreover, the fragment ions at *m*/*z* 280.26[LCB+H]^+^ revealed the length of the sphingoid long-chain base (LCB) (as depicted in Figure S3). Based on the above data, the structure of AcA was defined as (2R,3E)-N-((2S,3R,4E,8E)-3-hydroxy-9-methyl-1-(β-D-glucopyranosyloxy) 2-hydroxyheptadeca-4,8-dien-2-yl))icos-3-enamide and was named Aspercerebroside A (as depicted in Figure 1).

### 3.2. NO Production Assay

**Figure 2:**
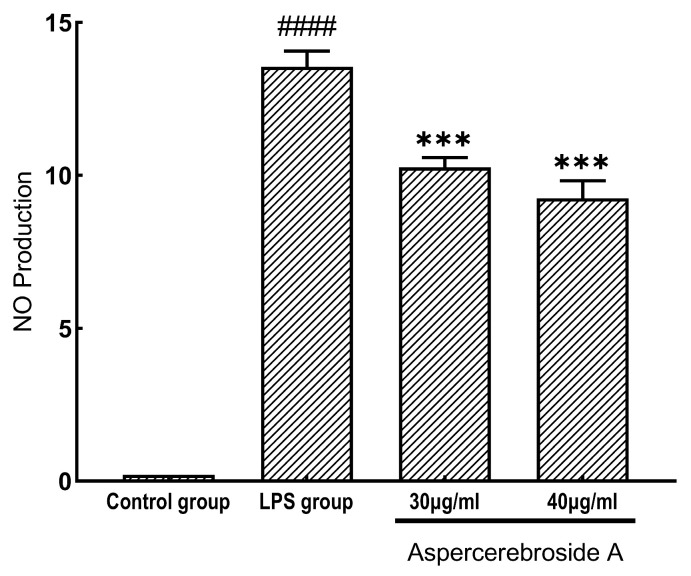
Inhibition of NO production by aspercerebroside A at concentrations of 30 μg/mL and 40 μg/mL in RAW 264.7 cells. Values represent the mean ± SD of three independent experiments. *** *p* < 0.001 (significant difference between the Aspercerebroside A treatment group and the LPS group). #### *p* < 0.001 (significant difference within the control group).

The inhibitory activity of the obtained aspercerebroside A on nitric oxide (NO) production was evaluated using an LPS-stimulated RAW 264.7 cell model. The NO release inhibition assay demonstrated that this compound exhibits significant anti-inflammatory effects at concentrations of 30 μg/mL and 40 μg/mL in Figure 2.

### 3.3. Single-Factor Experiments

#### 3.3.1. Influence of Carbon Source Types on Glycosylceramide Production

**Figure 3:**
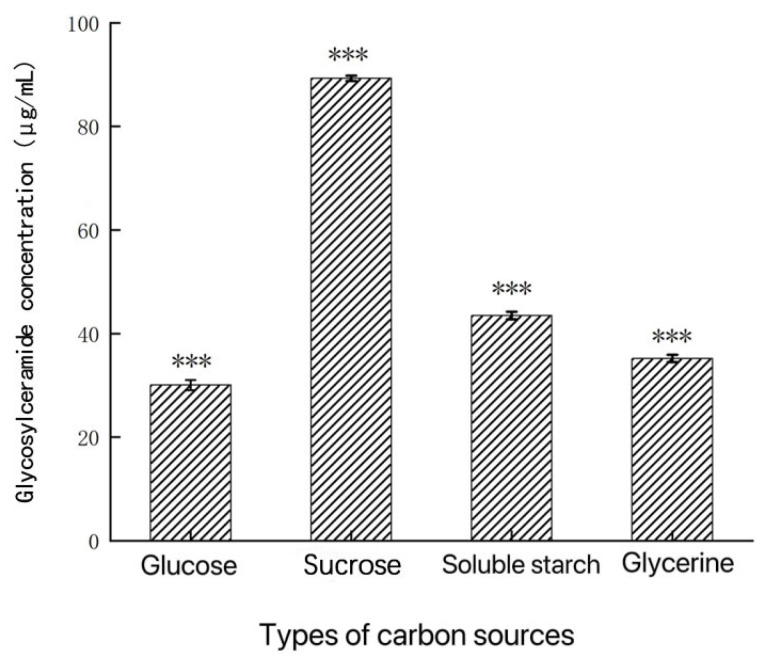
Influences of different carbon sources on glycosylceramide production from *Aspergillus* sp. ***: *p* < 0.001 (significant difference within the control group).

Carbon sources are essential nutrients in microbial culture media. They provide the carbon skeleton for cell growth and the synthesis of cellular products, as well as the energy required for cellular activities. As shown in Figure 3, when sucrose is used as the carbon source in the fermentation medium for *Aspergillus* sp., the glycosylceramide production is the highest, with a yield of 89.32 μg/mL. Soluble starch, glucose, and glycerol have a similar impact on glycosylceramide production, which may be related to the enzymatic system utilized by this strain in glycosylceramide biosynthesis. Therefore, based on the experimental results, sucrose was selected as the optimal carbon source for the fermentation medium.

**Figure 4:**
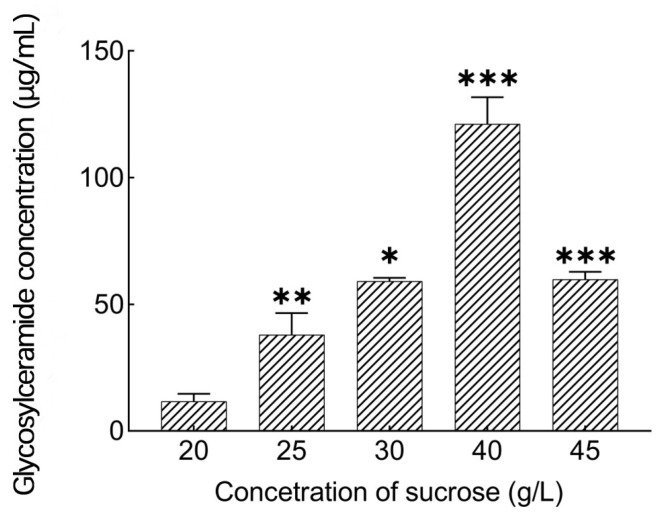
Influences of sucrose concentration on glycosylceramide production from *Aspergillus* sp.. *: *p* < 0.05, **: *p* < 0.01, ***: *p* < 0.001 (significant difference within the control group).

As shown in Figure 4, with the increase in sucrose concentration, glycosylceramide production first increased and then decreased. At 40 g/L, the maximum glycosylceramide production was 121 μg/mL. With the continuous increase in sucrose concentration, glycosylceramide production began to decline, which may have had a blocking effect.

#### 3.3.2. Influence of Nitrogen Source Types on Glycosylceramide Production

**Figure 5:**
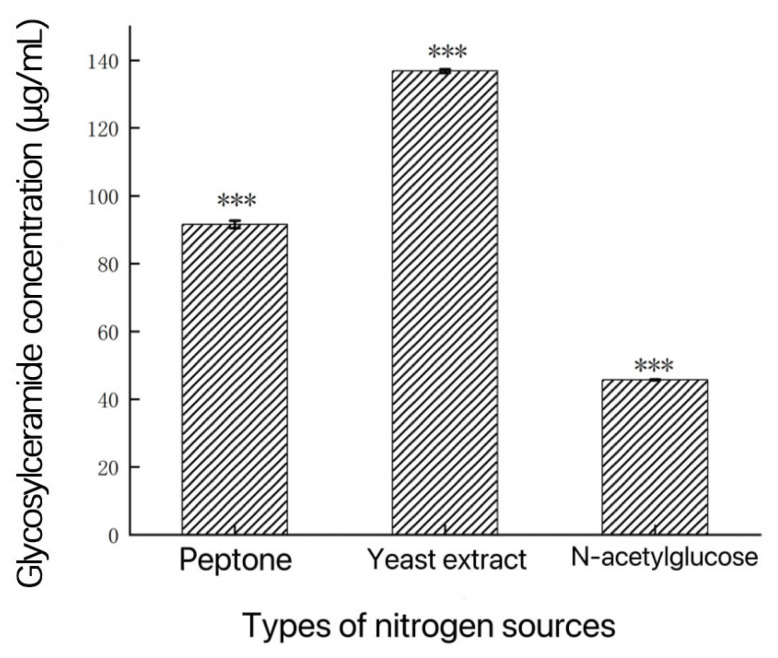
Influences of different nitrogen sources on glycosylceramide production from *Aspergillus* sp. ***: *p* < 0.001 (significant difference within the control group).

Nitrogen sources are essential components in the composition of living organisms. Their primary function is to provide the necessary nitrogen elements for the synthesis of amino acids, proteins, nucleic acids, and other essential compounds. As shown in Figure 5, in the single-factor experiments with different nitrogen sources, when yeast extract was used as the nitrogen source, the glycosylceramide production was the highest, reaching 136.80 μg/mL. Next was peptones, and then the lowest production was observed with N-acetylglucosamine as the nitrogen source. Therefore, yeast extract was selected as the nitrogen source for the culture medium based on these results.

**Figure 6:**
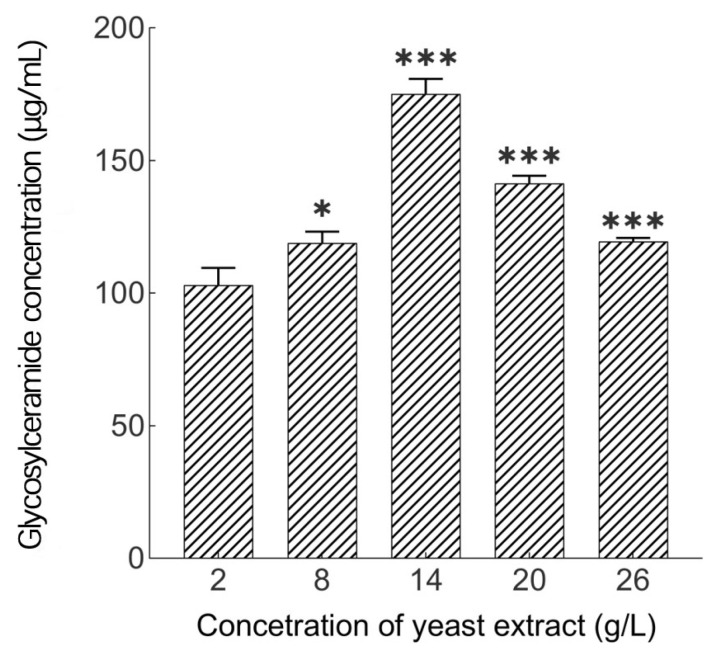
Influences of yeast extract concentration on glycosylceramide production from *Aspergillus* sp.*: *p* < 0.05, ***: *p* < 0.001 (significant difference within the control group).

As shown in Figure 6, Yeast extract powder has the greatest influence on glycosylceramide yield. When the concentration of yeast extract powder is 14 g/L, the glycosylceramide yield is the highest. When the concentration of yeast extract powder continues to increase, the glycosylceramide yield is inhibited and begins to decline, Therefore, the optimal concentration is 14 g/L.

#### 3.3.3. Influence of Culture Medium Salinity on Glycosylceramide Production

**Figure 7:**
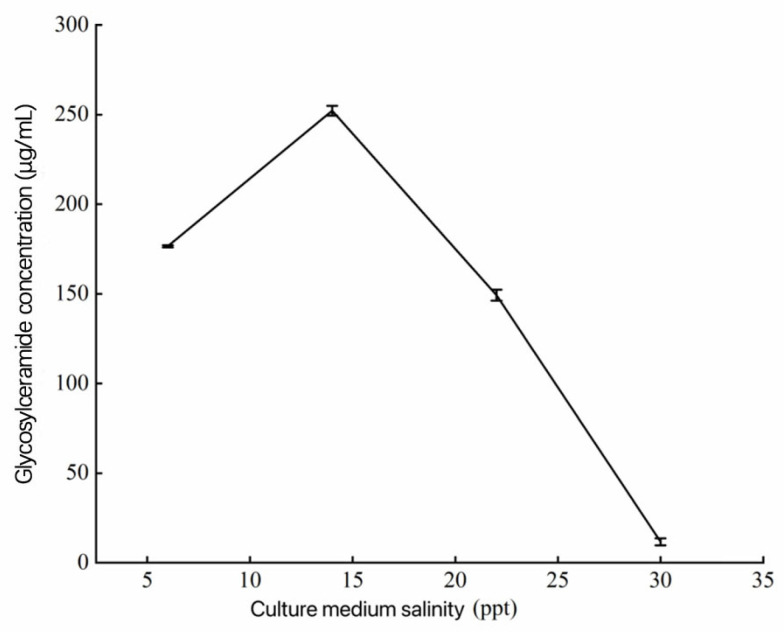
Influences of different nitrogen sources on glycosylceramide production from *Aspergillus* sp.

Marine microorganisms face a unique environment characterized by high salinity, which differs from that of terrestrial microorganisms. The addition of ecological sea salt to the culture medium not only helps replicate the components of seawater but also provides essential inorganic salts for microbial growth. Changes in culture medium salinity may affect the concentration of glycosylceramides in the secondary metabolites of *Aspergillus*. sp. As shown in Figure 7, when the culture medium salinity was 14 ppt, the marine *Aspergillus* fungus produced the maximum amount of glycosylceramides, reaching 245.46 μg/mL. However, as the salinity of the culture medium increased beyond this point, the concentration of glycosylceramides gradually decreased. When the culture medium salinity reached 30 ppt, the glycosylceramide concentration was at its minimum, at only 11.47 μg/mL.

### 3.4. Orthogonal Array Experiment

Based on the single-factor results of various influencing factors and their levels, *Aspergillus* sp. was cultured in a fermentation medium containing 45 g/L sucrose, 8 g/L yeast extract, and an appropriate amount of ecological sea salt. An orthogonal array L9 (34) was used according to the selected influencing factors and their levels. The influencing factors and their levels to be investigated are shown in Table 3. The orthogonal experimental table L9 (34) and the experimental results are presented in Table 4, while the variance analysis data processing can be found in Table 5.

From the intuitive analysis in Table 4 using the range analysis method, it can be seen that the most significant factor affecting the production of aspercerebroside A by marine-derived *Aspergillus* sp. is temperature, and the optimal combination for aspercerebroside A production is A3B3C3D3. The K values refer to the sum of the experimental data Y for each factor at every level. For instance, in this experiment, K1 for factor A represents the sum of the nervonic acid yield for all experimental groups at pH levels ranging from 6.2 to 6.4. R value stands for the range of factors, reflecting the degree of variation among different levels. By analyzing its magnitude, it is possible to make an initial ranking of the selected factors based on their differences. K1, K2, and K3 are the sum values corresponding to different factor levels. K1/9, K2/9, and K3/9 are the means of these sum values. The R value is calculated by subtracting the smallest mean value from the largest mean value.

Table 5, utilizing the variance analysis method, shows that the order of influence on aspercerebroside A production is temperature > time > initial salinity > initial pH value. All four factors have a highly significant impact on aspercerebroside A yield. Based on the analysis of the results, the discrepancy between the order of influence of initial salinity and initial pH value observed in the range analysis and variance analysis may be due to the closeness of their effects on aspercerebroside A production. Moreover, range analysis cannot estimate the inevitable errors that occur during experiments and the measurement of the results. Therefore, the results are considered more reliable based on variance analysis.

To validate these orthogonal results, marine-derived *Aspergillus* sp. was cultured under the optimized fermentation conditions from the best combination. The aspercerebroside A content obtained from this experiment was 436.86 μg/mL.

#### 3.4.1. Response Surface Optimization Results and Analysis

Using the sucrose concentration, yeast extract concentration, and initial salinity as independent variables, a three-factor, three-level response surface experiment was conducted through the Box–Behnken design method to analyze the interactive effects among various influencing factors. The response surface results are shown in Table 6.

The regression equation for the neuralamide concentration (Y) in relation to sucrose concentration (A), yeast extract concentration (B), and initial salinity (C), as obtained through data analysis using the Design-Expert 8.0.6.1 software, is as follows:

Y = 168.54 − 1.14A + 4.81B + 0.25C − 0.83AB + 0.70AC − 0.75BC − 1.96A^2^ − 2.81B^2^ − 4.73C^2^

#### 3.4.2. Variance Analysis

From Table 7, it can be observed that the *p*-value of the model is less than 0.0001, indicating that the regression equation model is highly significant. The lack-of-fit *p*-value is 0.4638, which is greater than 0.05, suggesting that errors due to random factors do not significantly affect the experimental results. The model’s determination coefficient (R^2^) is 0.9851, indicating that 98.51% of the response variation in neuralamide concentration is explained by the selected variables. The adjusted R^2^ (R^2^adj) is 0.9960, signifying that the model can explain 99.60% of the variation in neuralamide concentration response. Overall, the established model fits well and can be used to predict the concentration of neuralamide in fermentation production.

The analysis of variance (ANOVA) reveals that the first-order terms A and B have highly significant differences, indicating that the sucrose concentration and yeast extract concentration both have a highly significant impact on the concentration of neuralamide produced by the strain. The second-order terms A^2^, B^2^, and C^2^ exhibit highly significant differences, suggesting that the relationship between neuralamide concentration and sucrose concentration, yeast extract concentration, and initial salinity is not linear. The interaction terms AB, AC, and BC all have *p*-values greater than 0.05, indicating that the pairwise interactions between the three factors are not significant. Based on the F-values, the order of significance of the three selected variables in influencing the neuralamide production concentration is as follows: yeast extract concentration (B) > sucrose concentration (A) > initial salinity (C).

#### 3.4.3. RSM Analysis

**Figure 8:**
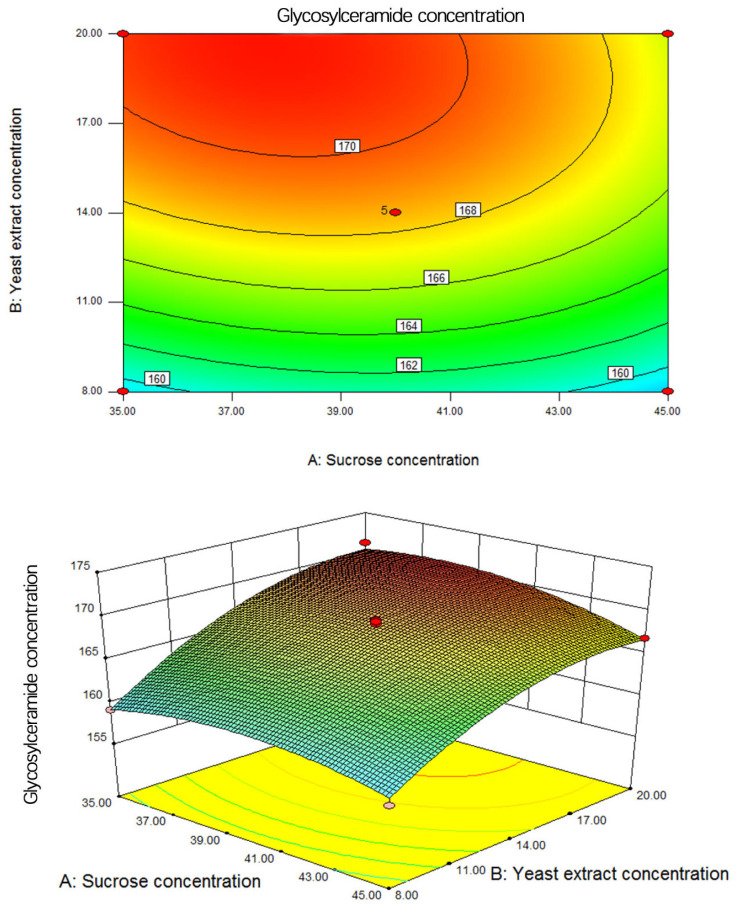
Contour and response surface diagram of sucrose concentration and yeast extract powder concentration on glycosylceramide concentration.

The interaction between sucrose concentration and yeast extract concentration is depicted in Figure 8. These figures include a contour plot and a response surface plot, with the culture medium salinity being held constant at 14 ppt. The contour plot predominantly showcases an elliptical shape, underscoring the substantial interaction between sucrose concentration and yeast extract concentration in their collective impact on glycosylceramide concentration. In the 3D surface plot, it becomes apparent that the surface representing sucrose concentration undergoes more gradual changes when compared to the surface representing yeast extract concentration. This observation suggests that the yeast extract concentration exerts a more pronounced influence on glycosylceramide concentration within the strain.

**Figure 9:**
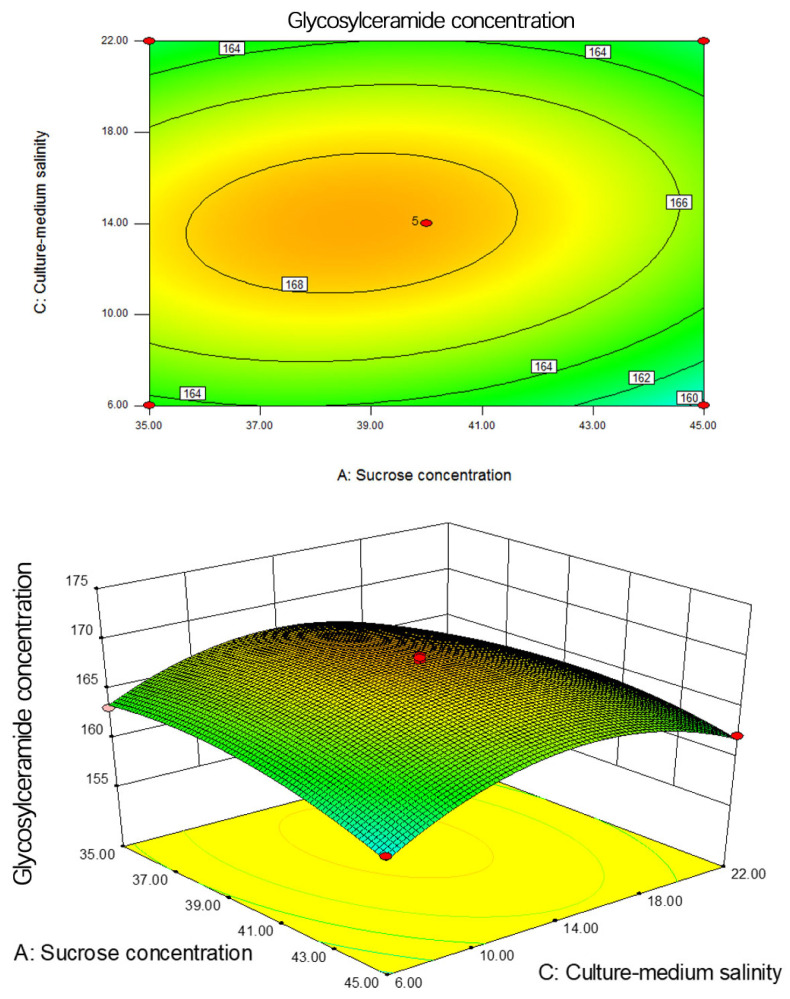
Contour and response surface diagram of sucrose concentration and culture-medium salinity on glycosylceramide concentration.

Furthermore, Figure 9 displays the contour plot and response surface plot for the interaction between sucrose concentration and initial salinity when yeast extract concentration is fixed at 14 g/L. The contour plot is elliptical in shape, indicating a significant interaction between sucrose concentration and initial salinity on neuralamide concentration. In the 3D surface plot, the initial salinity surface changes more gradually compared to the sucrose concentration surface, implying that sucrose concentration has a greater influence on the neuralamide concentration produced by the strain.

**Figure 10:**
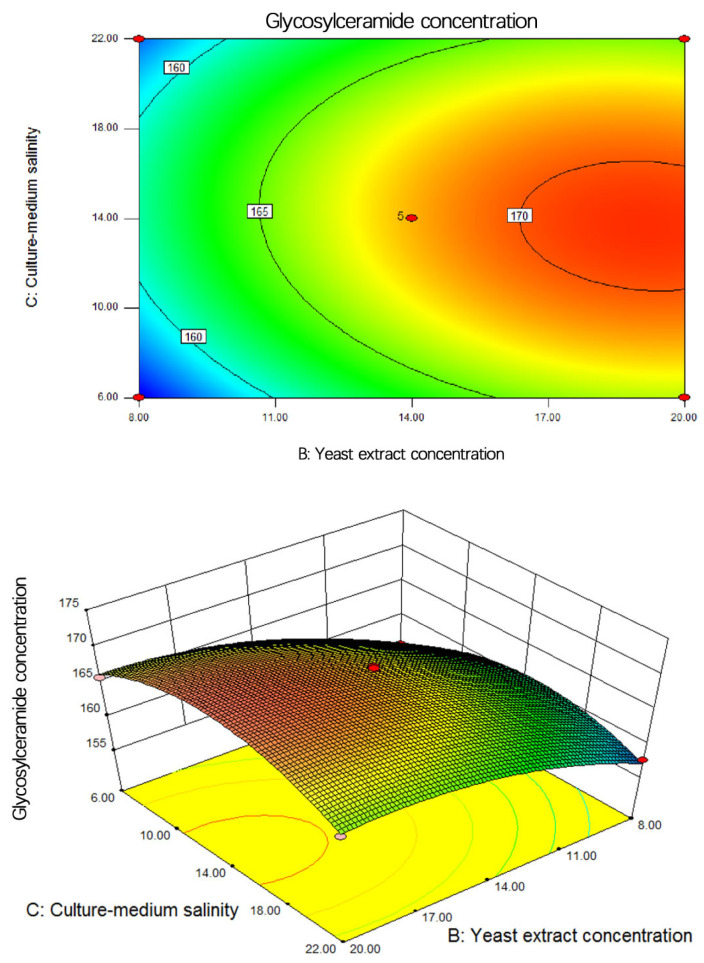
Contour and response surface diagram of yeast extract powder concentration and culture-medium salinity on glycosylceramide concentration.

The influence on neuralamide concentration is influenced by the strain. Figure 10 depicts a contour plot and a response surface plot illustrating the interaction between the yeast extract concentration and initial salinity, with the sucrose concentration fixed at 40 g/L. The contour plot displays an overall elliptical shape, indicating a strong interaction between yeast extract concentration and initial salinity concerning neuralamide concentration. In the 3D surface plot, the surface representing yeast extract concentration changes more significantly compared to the initial salinity surface, suggesting that the yeast extract concentration has a notably greater impact on the glycosylceramide production of the strain than sucrose concentration.

### 3.5. Verification Experiment Results

Based on the response surface analysis, the predicted optimal culture medium formula for producing glycosylceramides with *Aspergillus* sp. was determined as follows: a sucrose concentration of 37.47 g/L, yeast extract concentration of 19.66 g/L, and a culture medium salinity of 13.31 ppt. Under these conditions, the glycosylceramide concentration was predicted to be 171.084 µg/mL. In practical experiments, the model parameters were adjusted to a sucrose concentration of 37.5 g/L, yeast extract concentration of 19.7 g/L, and culture medium salinity of 13.3 ppt. Three parallel repeated experiments were conducted, resulting in glycosylceramide concentrations of 172.421 µg/mL, 171.976 µg/mL, and 170.612 µg/mL, with an average value of 171.670 µg/mL. These results closely aligned with the predicted values, indicating a strong model fit and high model credibility.

## 4. Discussion

It is worth noting that within *Aspergillus* sp., glycosylceramide compounds falling under the glycosylceramide class have been isolated [21]. These compounds exhibit similarities to AcA, sharing a molecular weight range between 760 and 780 [22]. This similarity indicates a resemblance between these glycosylceramide variants and underscores their association with aspercerebroside A within the glycosylceramide category. Such findings further emphasize the existence of analogous glycosylceramide compounds in the *Aspergillus* sp. genus, aligning with our identification of glycosylceramide (AcA) and supporting its classification within the broader glycosylceramide spectrum.

In marine environments, microorganisms harbor abundant resources, and yet the production of potentially valuable metabolites often demonstrates low yields during initial fermentation. Hence, the optimization of microbial fermentation processes has become pivotal to enhance metabolite production and fully exploit marine microbial resources. Typically, optimizing microbial fermentation conditions involves a blend of statistical and mathematical modeling techniques, as evidenced in previous studies. For instance, Wang Cong et al. [23] successfully optimized fermentation conditions for enhancing lincomycin D production by Streptomyces parvulus OUCMDZ-2554, resulting in a remarkable 3.6-fold increase in yield. Similarly, Dong Yanjuan et al. [24] optimized the fermentation conditions for elevating xylanase production by strain YS1069, achieving a remarkable five-fold increase in xylanase activity.

However, studies focusing on enhancing glycosylceramide fermentation yields have been relatively limited. Prior research primarily centered on the applications and extraction methods of glycosylceramides, such as elevating glycosylceramide production through Ganoderma lucidum fermentation or preparing glycosylceramides through emulsion fermentation. This study bridges a gap in this domain by augmenting glycosylceramide production through the optimization of culture medium specifically designed for marine-derived microorganisms.

During the optimization of conditions by selecting the initial pH value and initial salinity as variables, it was observed that the initially fixed salinity of the culture medium would fluctuate due to subsequent pH adjustments. Throughout the fermentation process, both salinity and pH dynamically changed. Comprehensive monitoring of salinity and acidity throughout the fermentation might identify the most suitable conditions for synthesizing glycosylceramides. The experimental findings revealed that at a temperature of 20 °C, the strain’s growth was notably inhibited compared to the standard temperature (25 °C), whereas at 30 °C, the growth accelerated significantly. Temperature was identified as a critical factor influencing glycosylceramide production, suggesting the potential to increase temperature moderately to enhance the strain’s metabolic capacity.

Simultaneously, throughout fermentation, time displayed a direct correlation with glycosylceramide accumulation. Identifying an optimal fermentation timepoint combined with an appropriate temperature could significantly enhance fermentation capability, optimizing the cost-effectiveness of glycosylceramide production. In summary, optimizing the fermentation of the marine-derived fungus Aspergillus sp. can be achieved by enhancing its own development and nutrient synthesis through adjustments in suitable culture medium components such as inorganic salts, the number of carbon and nitrogen source types, as well as regulating the fermentation liquid’s pH acidity and salinity and altering the fermentation temperature, duration, inoculum size, and speed to investigate their impact on metabolite production and content. Further exploration of the interaction between these factors could refine the fermentation conditions for optimal glycosylceramide synthesis.

## 5. Conclusions

This investigation explored the anti-inflammatory potential of AcA using LPS-induced RAW 264.7 cells, revealing significant inhibitory effects of Compound 1 on nitric oxide (NO) production in RAW 264.7 cells, particularly at concentrations of 30 µg/mL and 40 µg/mL. This discovery holds crucial importance in identifying potent active components derived from marine fungi.

In this study, a comprehensive investigation was conducted to determine the fermentation parameters for *Aspergillus* sp. derived from marine fungi. Initially, single-factor impact experiments were employed to explore the range and trends of carbon sources, nitrogen sources, initial pH values, salinity, temperature, and time fluctuations during fermentation. Subsequently, an orthogonal study was conducted, focusing on four influencing factors—initial pH value, initial salinity, temperature, and time—at three suitable levels, resulting in the optimal fermentation combination of A3B3C3D3. Experimental validation confirmed that the most suitable fermentation conditions for Aspergillus sp. were as follows: 45 g/L sucrose, 8 g/L yeast extract, initial salinity of 22 ppt (using ecological mineral salts to adjust fermentation medium), initial pH range of 8.2 to 8.4, and cultivation at 30 °C for 5 days, resulting in a glycosylceramide content of 436.86 μg/mL. Additionally, these optimized fermentation conditions resulting in a glycosylceramide content represent a significant finding in this research. Glycosylceramide production under such precise and enhanced conditions, previously sparsely discussed in the existing literature, emphasizes the novelty and importance of this study’s outcomes.

Furthermore, employing a combination of single-factor impact experiments and response surface optimization led to the successful determination of the optimal culture medium formulation for glycosylceramide production by Aspergillus sp. derived from marine fungi. This formulation consisted of 37.5 g/L sucrose, 19.7 g/L yeast extract, and a culture medium salinity of 13.3 ppt. Experimental validation demonstrated a close alignment between actual and predicted production values, affirming the high reliability of the established regression model.

In conclusion, The discovery of these specific fermentation parameters offers a valuable contribution to the field, particularly in elucidating optimal conditions for glycosylceramide biosynthesis in *Aspergillus* sp. derived from marine fungi. It not only expands the limited literature discussing these specific fermentation conditions but also paves the way for potential applications in industrial-scale glycosylceramide production.

## Figures and Tables

**Table 1:** Factors and levels for response surface experiments.

Factor	Level		
	−1	0	1
Soluble starch g/L	35	40	45
Yeast extract g/L	8	14	20
Culture-medium salinity ppt	6	14	22

**Table 2:** ^1^H and ^13^C NMR Data for 1 (500, 125 MHz,DMSO-d6, TMS, *δ* ppm).

No.	*δ*_C_	*δ*_H_ (*J* in Hz)	No.	*δ*_C_	*δ*_H_ (*J* in Hz)
1″	103.50	4.11, ^1^H, d(7.75)	1	68.60	3.97, m
2″	73.39	2.96, ^1^H, m			3.51, dd(10.4,3.8)
3″	76.54	3.14, m	2	52.90	3.78, m
4″	70.01	3.04, m	3	70.51	3.97, m
5″	76.88	3.08, m	4	130.91	5.36, ^1^H, dd(15.4,6.7)
6″	61.06	3.66, brdd(10.1,6.1)	5	130.91	5.67, ^1^H, dt(15.4,6.7)
1′	172.01	—	6	27.37	1.94, m
2′	71.91	4.30, brt(4.78) t	7	32.13	1.94, m
3′	129.05	5.43, ^1^H, dd(15.4,5.4)	8	123.5	5.09, ^1^H, brt(6.1)
4′	130.91	5.45, ^1^H, dt(15.4,5.4)	9	134.91	—
5′	31.63	1.94, m	10	39.10	1.94, m
6′-15′	29.00	1.24, m	11	27.29	1.23, m
16′	31.28	1.24, m	12–14	29.05	1.23, m
17′	22.08	1.24, m	15	28.69	1.23, m
18′	13.92	0.85, 3H, t(6.875)	16	22.08	1.23, m
NH	—	7.38, d(9.2)	17	13.92	0.85, ^3^H, t(6.875)
OH-2′	—	5.77, d(5)	18	15.72	1.54, ^3^H, s
OH-2″	—	4.98, d(4.6)	OH-3	—	4.97, d(4.2)
OH-4″	—	4.94, d(4.6)	OH-3″	—	4.92, d(5.0)
			OH-6″	—	4.52, t(5.9)

**Table 3:** Experimental factors of SPSS design and their levels.

Level	A (Initial pH Value)	B (Initial Salinity/ppt)	C (Temperature/°C)	D (Time/d)
1	6.2~6.4	6	20	3
2	7.2~7.4	14	25	4
3	8.2~8.4	22	30	5

**Table 4:** SPSS design table and experimental results.

Number	A (Initial pH Value)	B (Initial Salinity/ppt)	C (Temperature/°C)	D (Time/d)	Glycosylceramide Concentration Group 1 (μg/mL)	Glycosylceramide Concentration Group 2 (μg/mL)	Glycosylceramide Concentration Group 1 (μg/mL)
1	1	1	1	1	28.62	27.06	21.44
2	1	2	3	2	193.07	177.85	187.81
3	1	3	2	3	201.87	229	211.22
4	2	1	3	3	209.74	178.74	185.28
5	2	2	2	1	73.62	83.87	76.82
6	2	3	1	2	74.82	90.04	114.03
7	3	1	2	2	169.61	139.41	171.84
8	3	2	1	3	122.38	139.19	141.14
9	3	3	3	1	177.40	178.04	215.03
K1	1277.94	1131.96	758.72	881.9			
K2	1087.18	1195.75	1279.11	1318.48			
K3	1454.04	1491.45	1703.18	1618.78			
K1/9	141.99	125.77	84.3	97.99			
K2/9	120.80	132.86	142.12	146.5			
K3/9	161.56	165.72	189.24	179.86			
R	40.76	39.94	104.94	81.88			

**Table 5:** Analysis of variance.

Source of Variance	Type III Square Sum	Df	the Average Square	F Value	Significance
A	7480.994	2	3740.497	18.178	*p* < 0.05
B	8175.582	2	4087.791	19.866	*p* < 0.05
C	50,737.610	2	25,368.805	123.287	*p* < 0.05
D	30,510.160	2	15,255.080	74.137	*p* < 0.05
Error	3703.852	18	205.770		

F0.05(2,18) = 3.555, F0.01(2,18) = 6.01.

**Table 6:** Response surface design test and results.

Test Number	Level			Glycosylceramide Concentration (μg/mL)
	Sucrose Concentration (A)	Concentration of Yeast Extract Powder (B)	Salinity of Medium (C)	
1	0	−1	−1	155.6
2	0	0	0	168.4
3	1	−1	0	157.9
4	1	0	1	162.2
5	1	0	−1	160.1
6	0	1	−1	165.8
7	0	−1	1	157.7
8	−1	0	1	161.9
9	−1	−1	0	159.1
10	0	1	1	164.9
11	1	1	0	166.8
12	0	0	0	169.1
13	0	0	0	167.1
14	0	0	0	168.8
15	−1	1	0	171.3
16	−1	0	−1	163.2
17	0	0	0	169.3

**Table 7:** Regression model analysis of variance.

Source of Variation	Quadratic Sum	Degree of Freedom		Mean Square	F-Value	*p*-Value
Model	360.73	9		40.08	51.53	<0.0001
Concentration of sucrose (A)	10.35	1		10.35	13.31	0.0082
Concentration of yeast extract (B)	185.28	1		185.28	238.22	<0.0001
Salinity of medium (C)	0.50	1		0.50	0.64	0.4490
AB	2.72	1		2.72	3.50	0.1035
AC	1.96	1		1.96	2.52	0.1564
BC	2.25	1		2.25	2.89	0.1328
A^2^	16.13	1		16.13	20.74	0.0026
B^2^	33.19	1		33.19	42.67	0.0003
C^2^	94.30	1		94.30	121.24	<0.0001
Residual error	5.44	7		0.78		
Out of fit term	2.39	3		0.80	1.05	0.4638
Net error	3.05	4		0.76		
Total deviation	366.18	16				
R^2^ = 0.9851			R^2^adj = 0.9960			

Note: *p* < 0.01 indicates a very significant difference; *p* < 0.05 indicates a significant difference.

## Data Availability

All data are available in the manuscript and in the Supplementary Information.

## Supplementary Materials

The following supporting information can be downloaded at: https://www.mdpi.com/article/10.3390/metabo14020099/s1. Figure S1: ^1^H NMR spectrum of 1, Figure S2: ^13^C NMR spectrum of 1, Figure S3: mass spectrum of 1, Figure S4: fungal culture on YM medium, Figure S5: glycosylceramide standard curve, Figure S6: influences of different carbon sources, Figure S7: influences of different nitrogen sources, Figure S8: influences of different nitrogen sources, Table S1: response surface design test and results, Table S2: regression model analysis of variance, Figure S9: contour and response surface diagram of sucrose concentration and yeast extract powder concentration, Figure S10: contour and response surface diagram of sucrose concentration and culture-medium salinity, Figure S11: contour and response surface diagram of yeast extract powder concentration and culture-medium salinity, Figure S12: contour and response surface diagram of sucrose concentration and yeast extract powder concentration on glycosylceramide concentration, Figure S13: contour and response surface diagram of sucrose concentration and culture-medium salinity on glycosylceramide concentration, Figure S14: contour and response surface diagram of yeast extract powder concentration and culture-medium salinity on glycosylceramide concentration.
