# Supplementary material for: Optimization of Fermentation Process for New Anti-Inflammatory Glycosylceramide Metabolite from Aspergillus sp
Source: Metabolites. 2024 Jan 31;14(2):99. doi: 10.3390/metabo14020099 (PMC10890386; doi:10.3390/metabo14020099)
Supplement: Supplementary file 1 [file metabolites-14-00099-s001.zip › metabolites-2790984-supplementary.pdf]

SUPPLEMENTARY MATERIAL

Contents

Figure S1. ^{1}H NMR spectrum of **1**.
Figure S2. ^{13}C NMR spectrum of **1**.
Figure S3. Mass spectrum of **1**.
Figure S4. Fungal culture on YM medium.
Figure S5. Glycosylceramide standard curve.
Figure S6. Influences of different carbon sources.
Figure S7. Influences of different nitrogen sources.
Figure S8. Influences of different nitrogen sources.
Table 1. Response surface design test and results.
Table 2. Regression model analysis of variance.
Figure S9. Contour and response surface diagram of sucrose concentration and yeast extract powder concentration**.**
Figure S10. Contour and response surface diagram of sucrose concentration and Culture-medium salinity.
Figure S11. Contour and response surface diagram of yeast extract powder concentration and Culture-medium salinity.
Figure S12. Contour and response surface diagram of sucrose concentration and yeast extract powder concentration on glycosylceramide concentration.
Figure S13. Contour and response surface diagram of sucrose concentration and Culture-medium salinity on glycosylceramide concentration.
Figure S14. Contour and response surface diagram of yeast extract powder concentration and Culture-medium salinity on glycosylceramide concentration.

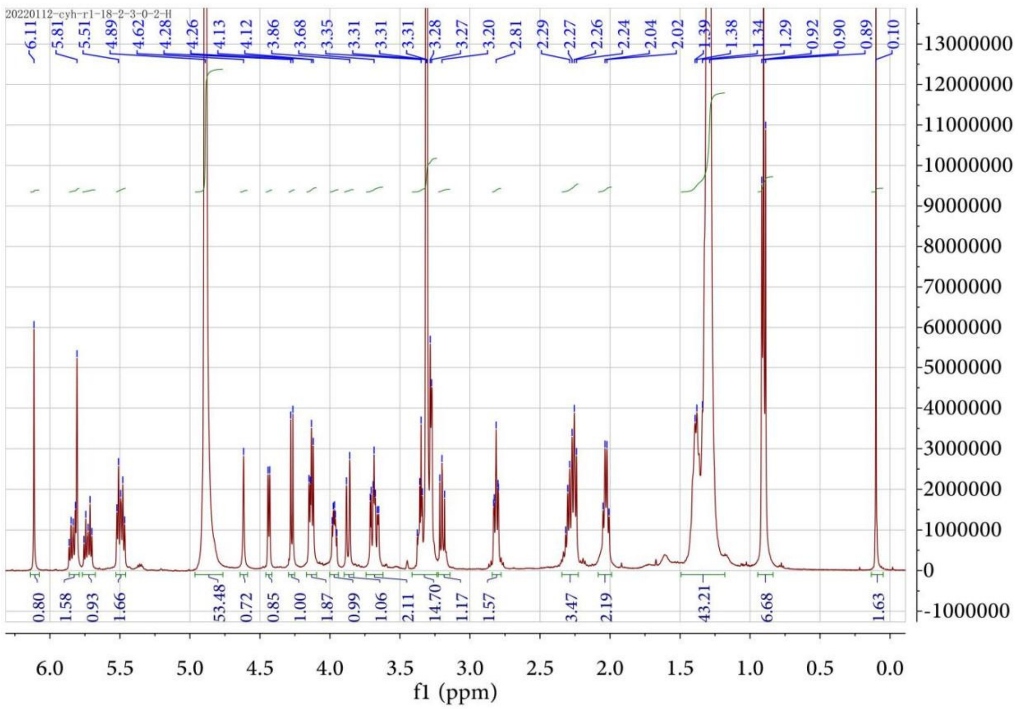

Figure S1. ^{1}H NMR spectrum of **1** (500 MHz, DMSO-d_6).

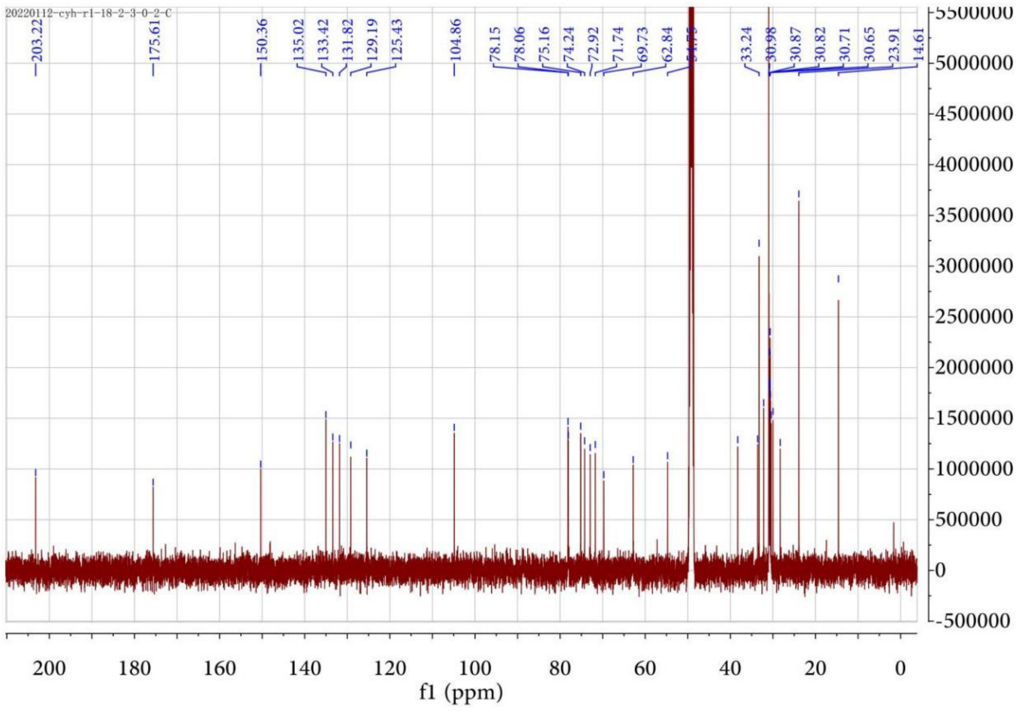

Figure S2. ^{13}C NMR spectrum of **1** (125 MHz, DMSO-d_6).

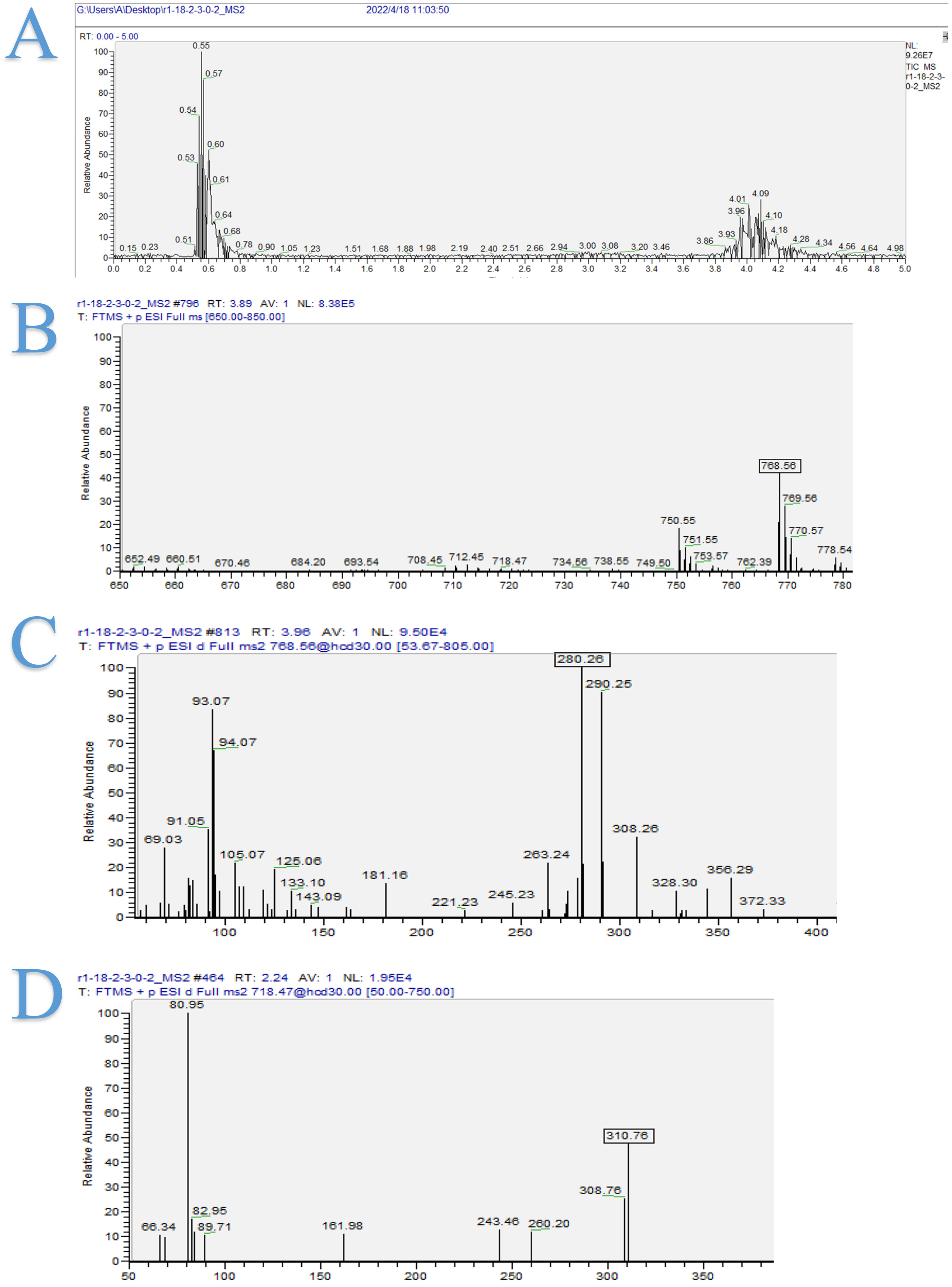

Figure S3. Mass spectrum of **1.**

A:The total ion current of 1. B:The total mass of 1 at m/z 768.56[M+H]$^+$

C:The LCB mass of 1 at m/z 280.26[LCB+H]$^+$

D:The FAB mass of 1 at m/z 310.76 [FAB+H]$^+$

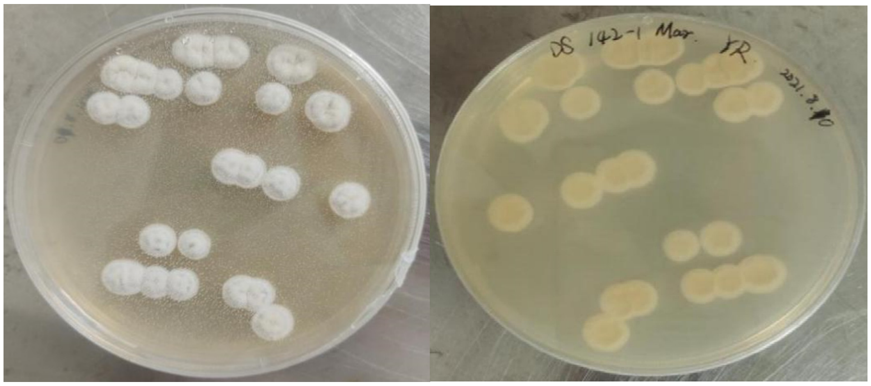

Figure S4. Fungal culture on YM medium.

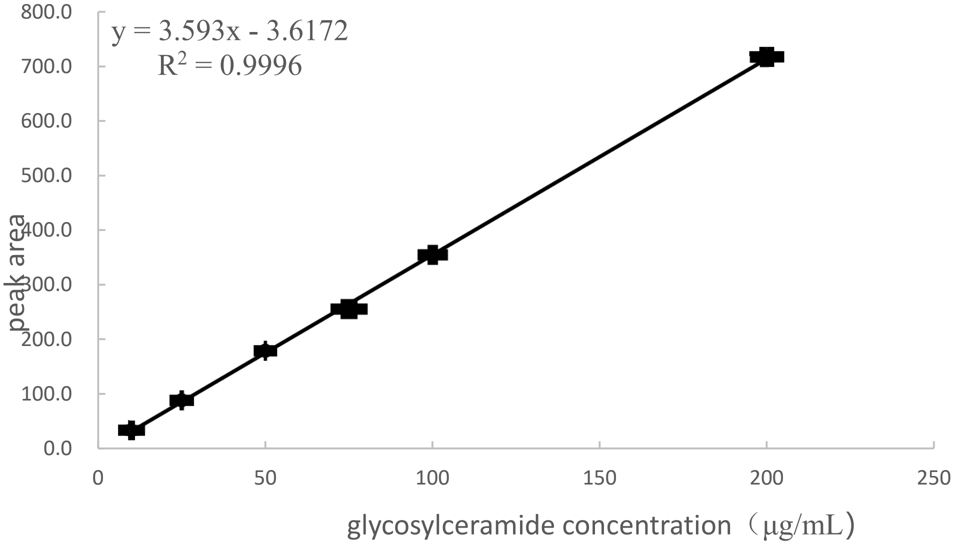

Figure S5. Glycosylceramide standard curve.

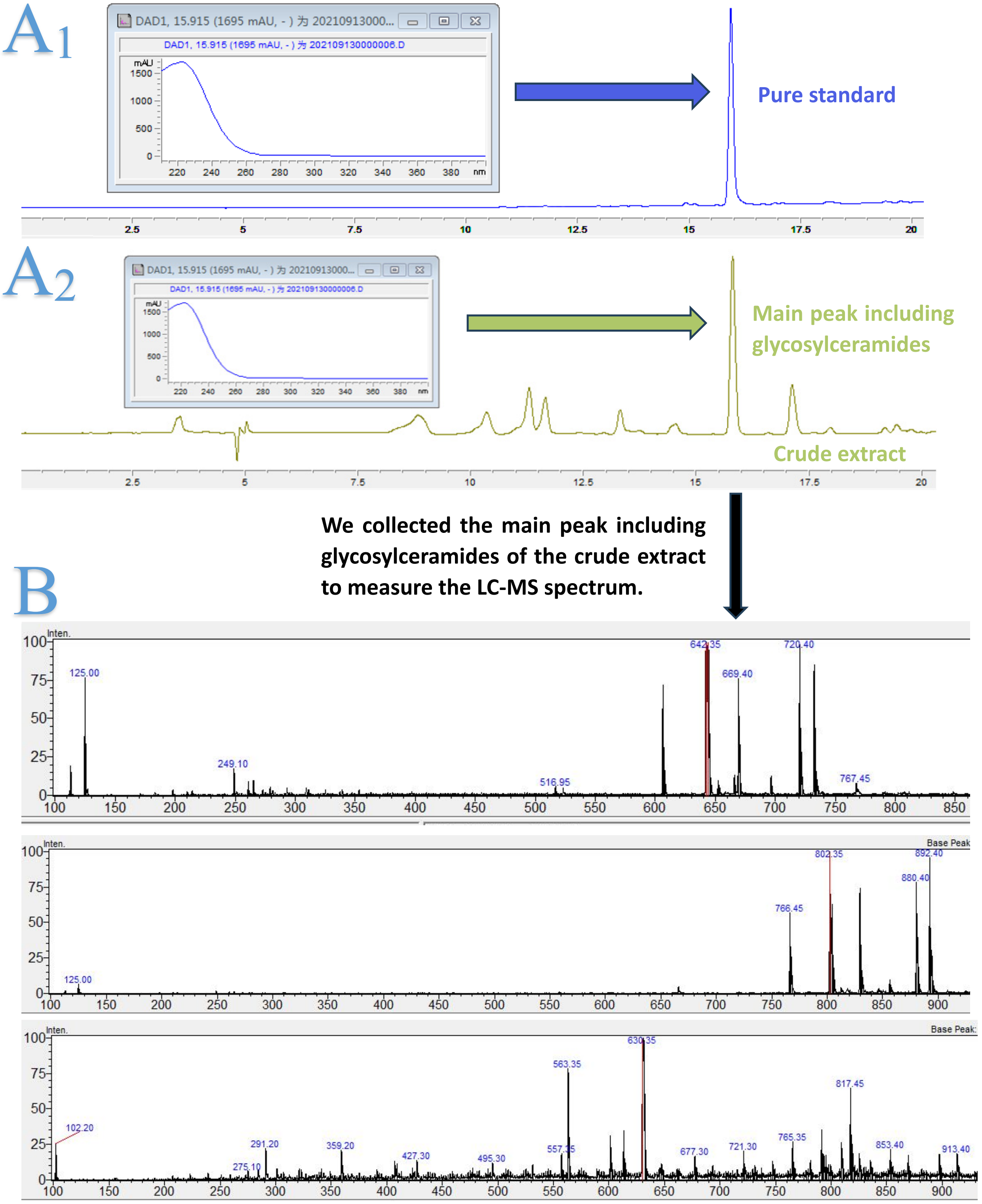

Figure S6. HPLC and LC-MS of crude extract and **1.**

A1:HPLC of **1.** A2:HPLC of the crude extract from *Aspergillus* sp.

B: LC-MS of the main peak including glycosylceramides of the crude extract.

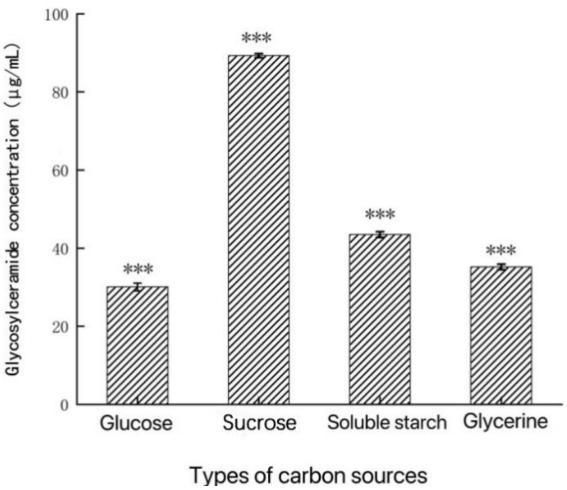

Figure S7. Influences of different carbon sources on glycosylceramide production from *Aspergillus* sp.

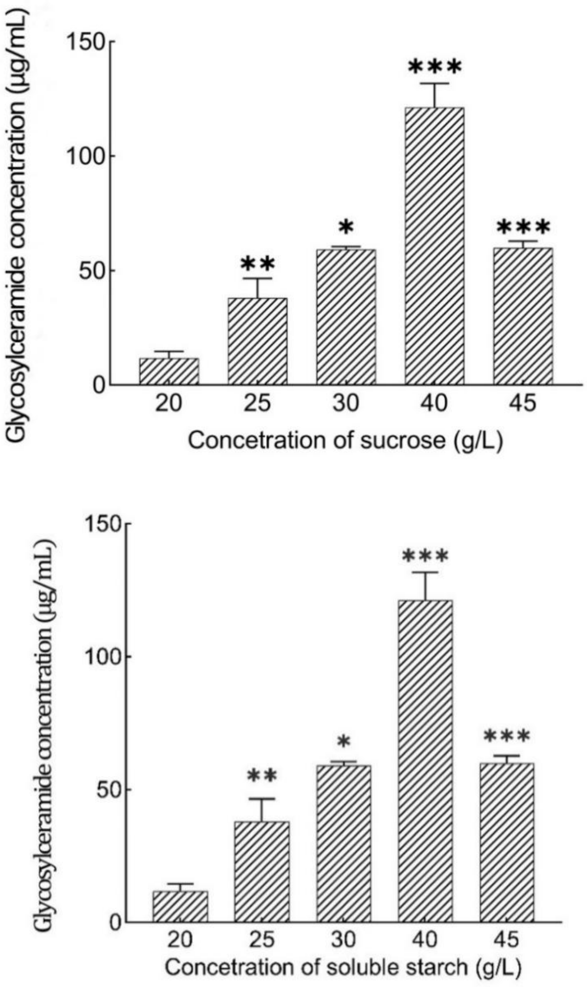

Figure S8. Influences of sucrose concentration on glycosylceramide production from *Aspergillus* sp.

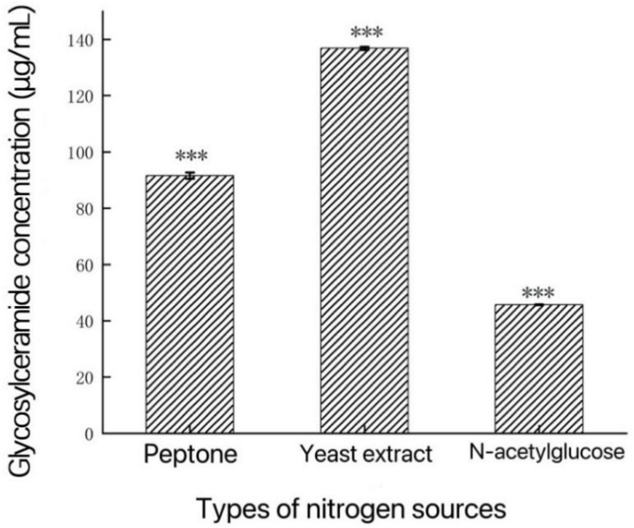

Figure S9. Influences of different nitrogen sources on glycosylceramide production from *Aspergillus* sp.

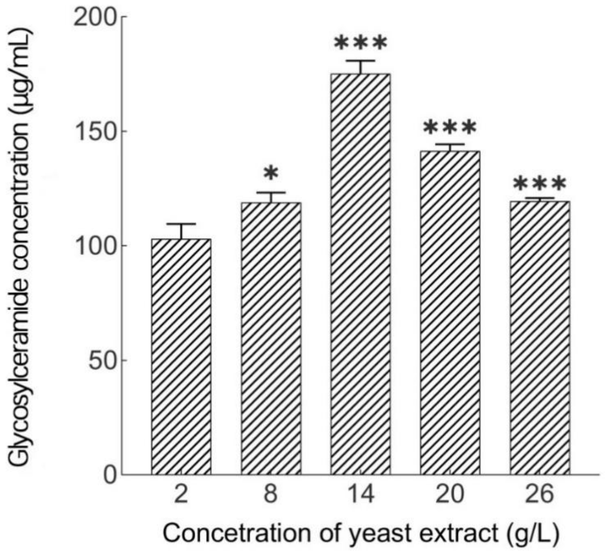

Figure S10. Influences of yeast extract concentration on glycosylceramide production from *Aspergillus* sp.

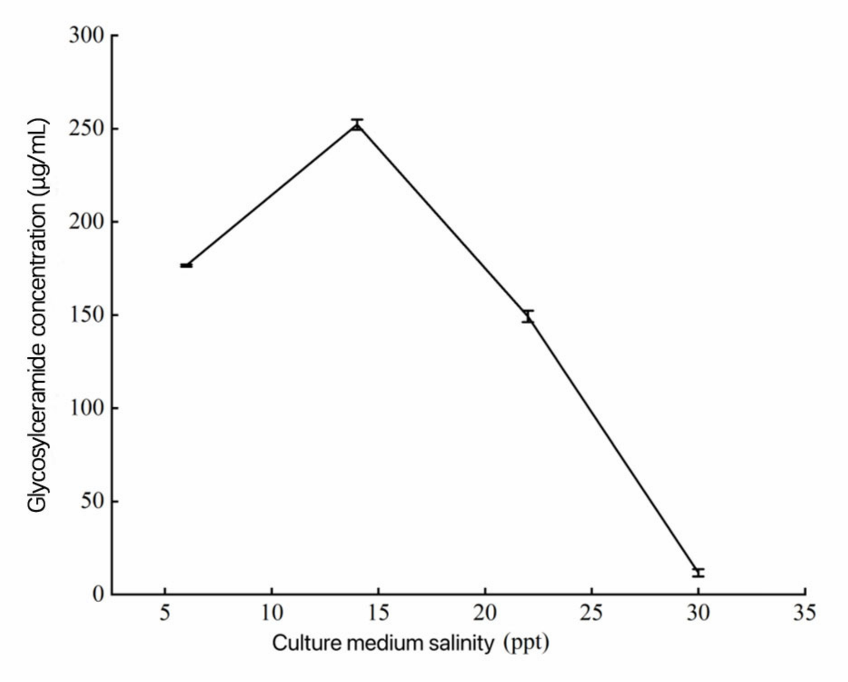

Figure S11. Influences of different nitrogen sources on glycosylceramide production from *Aspergillus* sp.

Table 1. Response surface design test and results

Test number	level			Glycosyl-ceramide concentration（μg/mL)
	Sucrose concentration（A）	Concentration of yeast extract powder（B）	Salinity of medium（C）	
1	0	-1	-1	155.6
2	0	0	0	168.4
3	1	-1	0	157.9
4	1	0	1	162.2
5	1	0	-1	160.1
6	0	1	-1	165.8
7	0	-1	1	157.7
8	-1	0	1	161.9
9	-1	-1	0	159.1

10	0	1	1	164.9
11	1	1	0	166.8
12	0	0	0	169.1
13	0	0	0	167.1
14	0	0	0	168.8
15	-1	1	0	171.3
16	-1	0	-1	163.2
17	0	0	0	169.3

The regression equation for the neuralamide concentration (Y) in relation to sucrose concentration (A), yeast extract concentration (B), and initial salinity (C), as obtained through data analysis using Design-Expert 8.0.6.1 software, is as follows:

$Y=168.54-1.14A+4.81B+0.25C-0.83AB+0.70AC-0.75BC-1.96A^2-2.81B^2-4.73C^2$

Table 2. Regression model analysis of variance

Soruce of variation	Quadratic sum	Degree of freedom	Mean square	F-value	P-value
Model	360.73	9	40.08	51.53	＜0.0001
Concentration of sucrose（A）	10.35	1	10.35	13.31	0.0082
Concentration of yeast extract（B）	185.28	1	185.28	238.22	＜0.0001
Salinity of medium（C）	0.50	1	0.50	0.64	0.4490
AB	2.72	1	2.72	3.50	0.1035
AC	1.96	1	1.96	2.52	0.1564
BC	2.25	1	2.25	2.89	0.1328
A^2	16.13	1	16.13	20.74	0.0026
B^2	33.19	1	33.19	42.67	0.0003
C^2	94.30	1	94.30	121.24	＜0.0001

Residual error	5.44	7	0.78		
Out of fit term	2.39	3	0.80	1.05	0.4638
Net error	3.05	4	0.76		
Total deviation	366.18	16			
R^2=0.9851 R^2adj=0.9960					

Note: P<0.01 indicates a very significant difference; P<0.05 indicates a significant difference

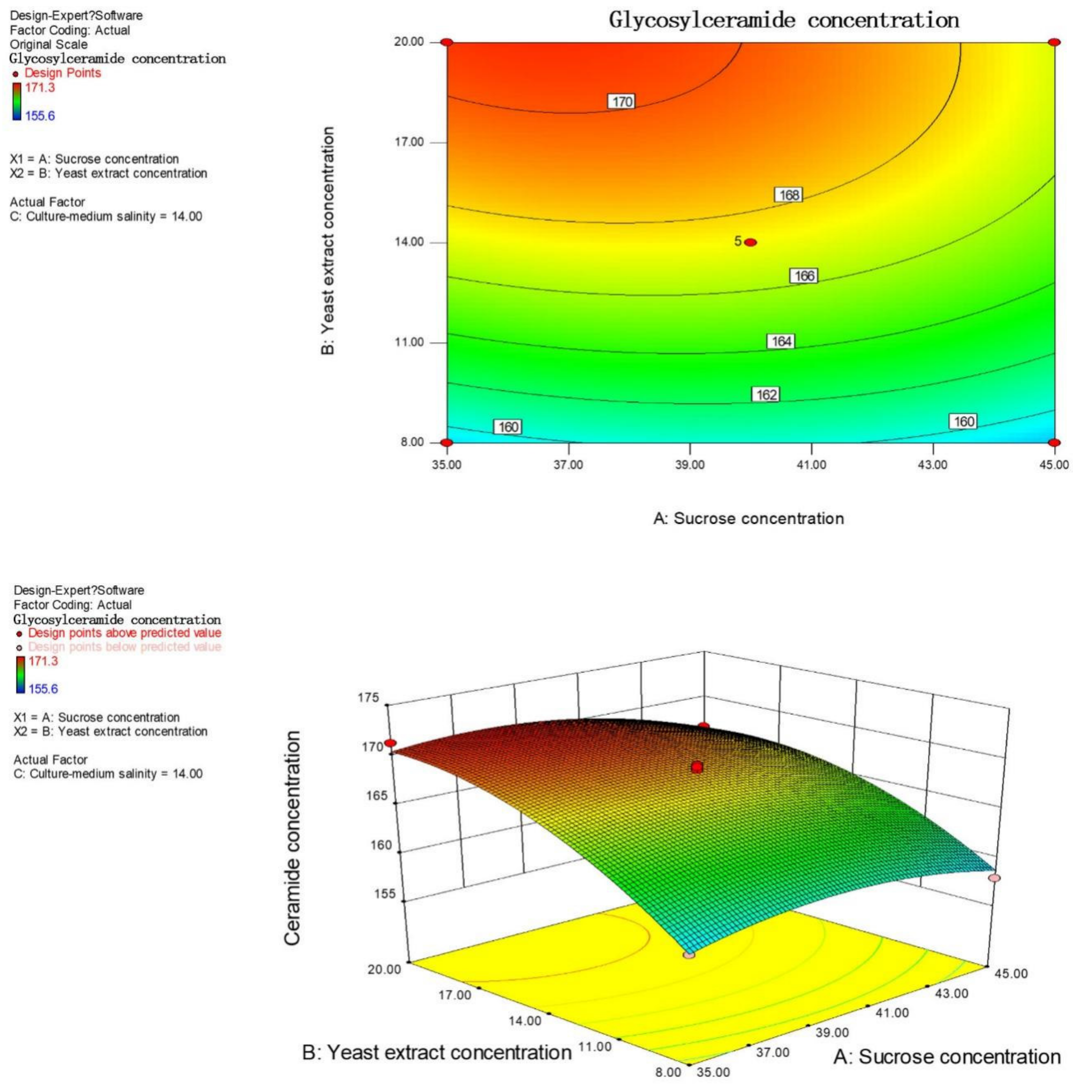

Figure S12. Contour and response surface diagram of sucrose concentration and yeast extract powder concentration on glycosylceramide concentration.

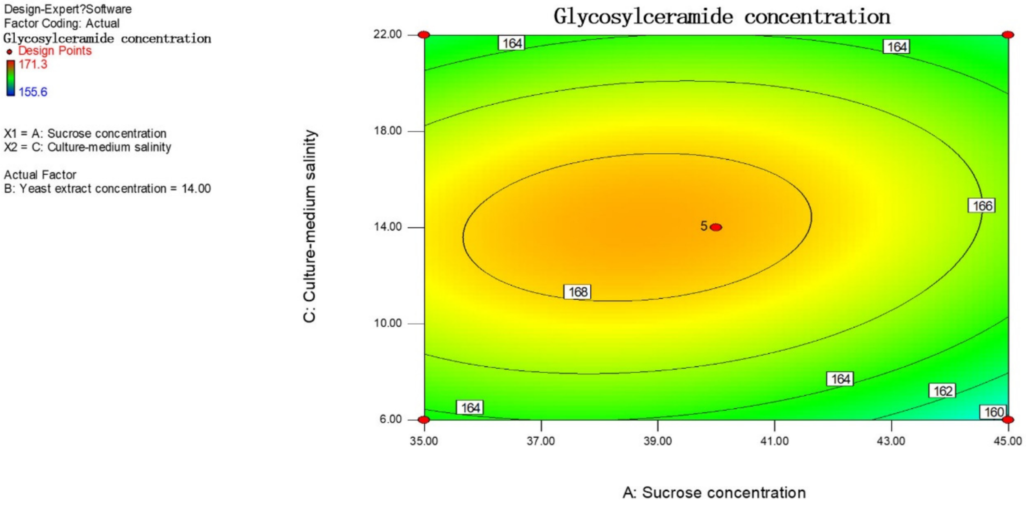

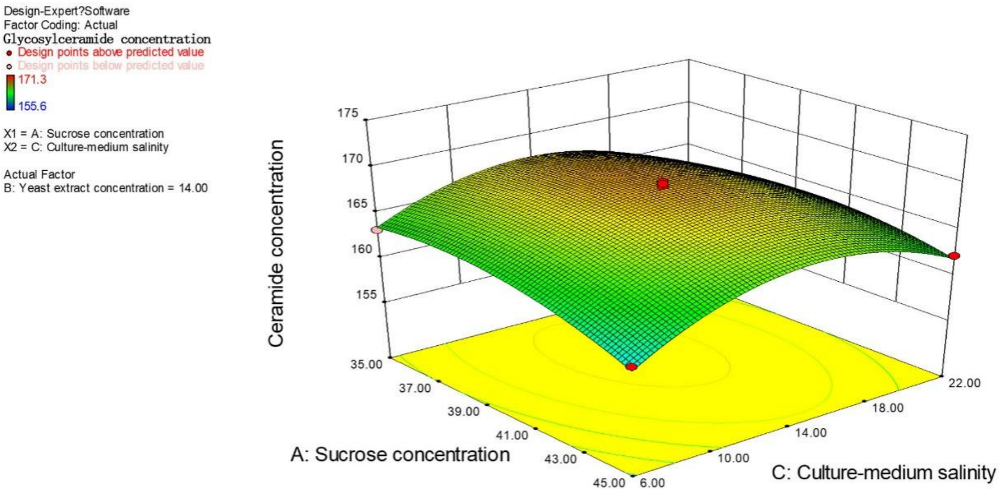

Figure S13. Contour and response surface diagram of sucrose concentration and Culture-medium salinity on glycosylceramide concentration.

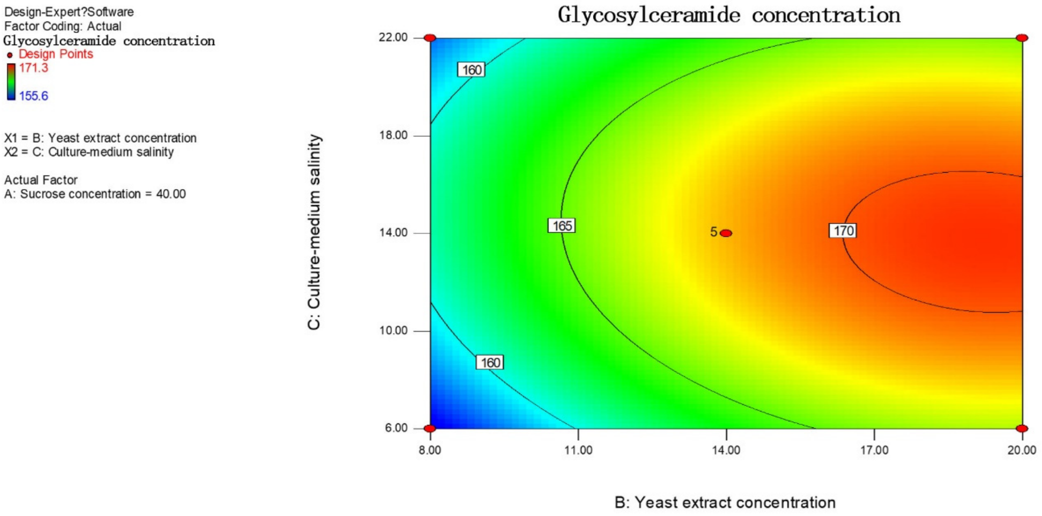

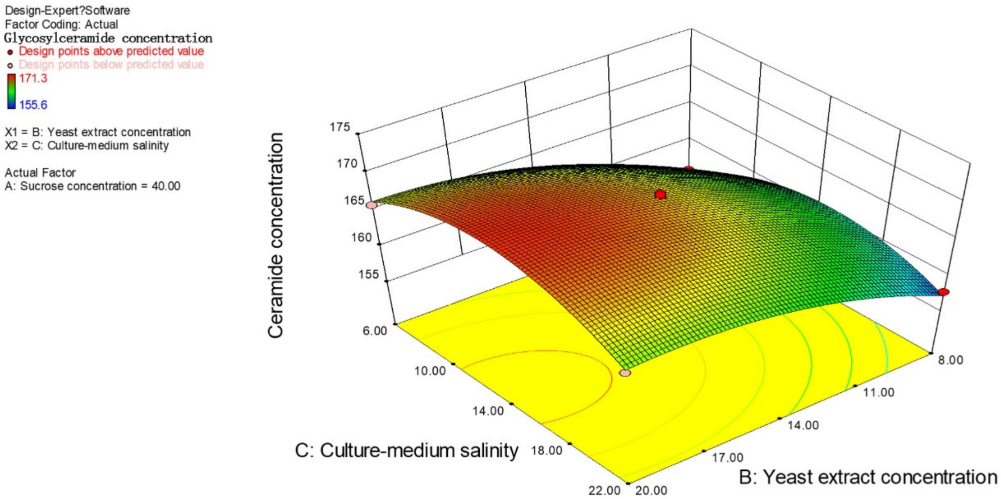

Figure S14. Contour and response surface diagram of yeast extract powder concentration and Culture-medium salinity on glycosylceramide concentration.
